# Erosive oral lichen planus successfully treated with JAK3/TEC inhibitor ritlecitinib: A case report

**DOI:** 10.1016/j.jdcr.2025.05.017

**Published:** 2025-06-16

**Authors:** Abigail Katz, Raphaella Lambert, Areeba Ahmed, Deep Patel, Gabriela Soto-Canetti, Jordan Talia, Jonas A. Adalsteinsson

**Affiliations:** a Department of Dermatology, Icahn School of Medicine at Mount Sinai, New York, New York; b Department of Dermatology, Northwestern University Feinberg School of Medicine, Chicago, Illinois; c Faculty of Medicine, University of Iceland, Reykjavík, Iceland; d Department of Dermatology, Landspitali University Hospital, Reykjavík, Iceland

**Keywords:** bullous skin diseases, erosive oral lichen planus, Janus kinase 3 inhibition, ritlecitinib

## Introduction

Oral lichen planus (OLP) is a chronic inflammatory condition affecting the oral mucosa.^1^, ^2^, ^3^ OLP is characterized by desquamative gingivitis, painful erosions, and reticular white lesions known as Wickham striae.^2^, ^3^, ^4^ The pathogenesis of OLP remains incompletely understood; however, an aberrant cytotoxic CD8+ T cell immune response has been implicated.^1^^,^^3^^,^^5^ T helper 1 (Th1) and Th17 inflammatory pathways are hypothesized to be key drivers of OLP, with frequent observation of high levels of -ɣ, mediated via Janus kinase (JAK) signaling.^1^^,^^2^^,^^5^, ^6^, ^7^, ^8^

Management of OLP begins with topical corticosteroids or calcineurin inhibitors, but severe cases may require systemic therapies.^2^^,^^8^^,^^9^ Traditional systemic treatments, like glucocorticoids and immunosuppressants, have significant side effects and a high risk of relapse upon discontinuation.^3^ Recent interest has shifted toward targeted systemic therapies, including JAK inhibitors, for their ability to selectively modulate key immune pathways.^5^

Here, we present a case of erosive OLP successfully managed with ritlecitinib, a JAK3/TEC kinase inhibitor, highlighting the potential of JAK3 inhibition as a novel therapeutic approach for refractory OLP.

### Case report

**Fig 1:**
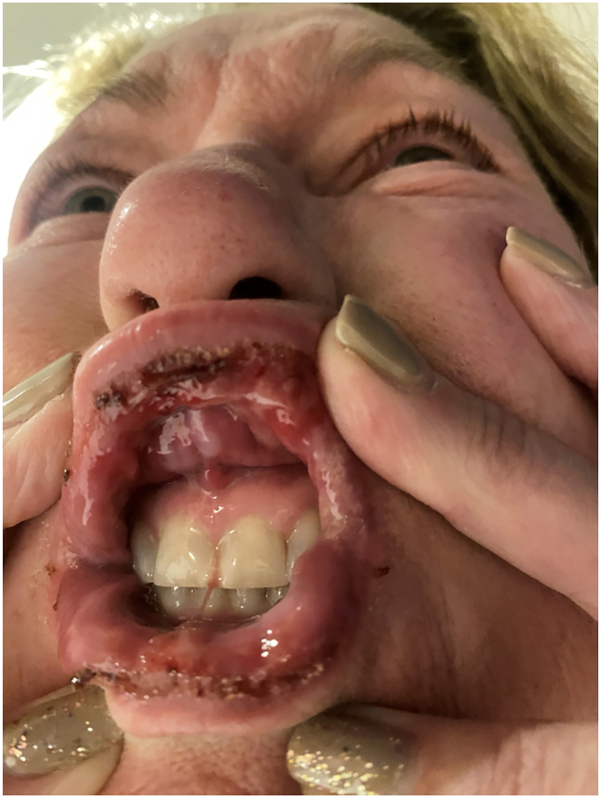
Baseline photo of the oral mucocutaneous lesions at the patient’s first visit at our practice.

**Fig 2:**
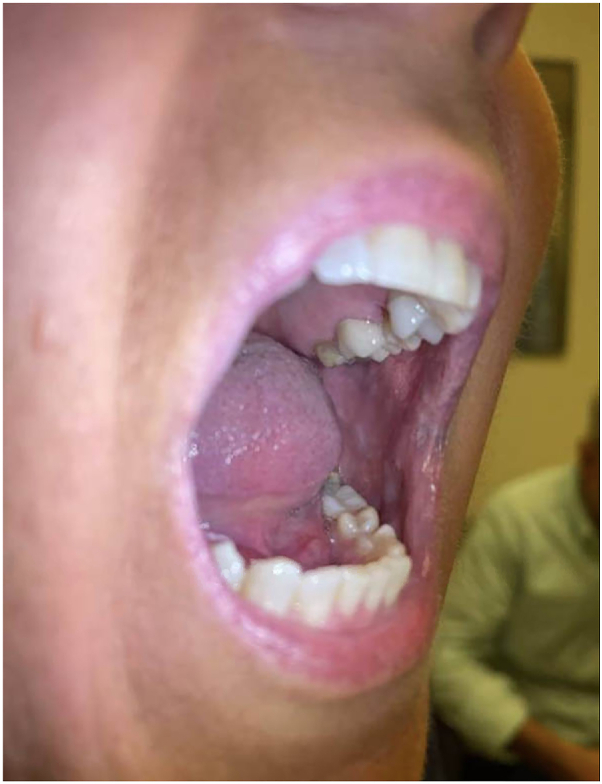
Oral lesions after 4 weeks of treatment with ritlecitinib. Faint Wickham striae still present, confirming the diagnosis of OLP. *OLP*, oral lichen planus.

A 60-year-old woman presented with an 18-month history of refractory oral ulcers and difficulty eating and drinking following a dental procedure (Fig 1). Initial workup was notable for negative hepatitis serologies and a prior biopsy revealed squamous mucosa with ulceration, nonspecific inflammation, and reactive epithelial changes, with negative immunostains for herpes simplex virus type 1 or 2 and fungus and a negative direct immunofluorescence (DIF). Examination revealed desquamative gingivitis involving the lips and buccal mucosa. The patient was on prednisone 2.5 mg daily, doxycycline 100 mg twice daily, and dexamethasone 0.05% oral rinse for over a year with minimal improvement and symptom recurrence upon steroid tapering. Given her selective improvement with prednisone and clinical findings, the differential included pemphigus vulgaris, OLP, mucous membrane pemphigus, and reactive infectious mucocutaneous eruption. Further diagnostic workup with indirect immunofluorescence, enzyme-linked immunosorbent assays, desmoglein antibodies 1 and 3 (Dsg 1/3), and bullous pemphigoid antibodies 180/230 was negative. Wickham striae on follow-up confirmed OLP (Fig 2).^3^^,^^4^

**Fig 3:**
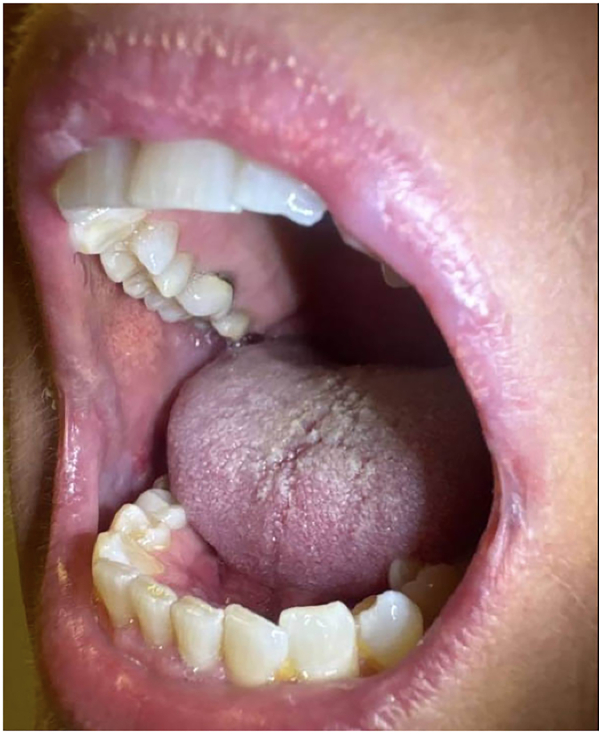
Oral mucocutaneous lesions significantly improved after 3 months of ritlecitinib.

Ritlecitinib 50 mg daily was subsequently added to the patient’s regimen, and she noted symptomatic improvement within 48 hours. She demonstrated clinical improvement after 7 days on ritlecitinib and tapered off prednisone and dexamethasone-swish by day 14. Ritlecitinib was tapered to 50 mg every other day, with continued improvement (Fig 2). However, further tapering to 50 mg every 3 days led to flares, necessitating a return to daily dosing alongside dexamethasone-swish. Symptomatic improvement was observed shortly after resuming this regimen (Fig 3). For the first time in 2 years, the patient is now completely off prednisone with adequate, long-term control of her disease and she has not experienced any significant lab abnormalities while on ritlecitinib.

## Discussion

This case demonstrates a novel usage of rilecitinib for the management of ulcerative OLP. Our patient had intractable severe OLP on presentation, unresponsive to any topicals or oral agents besides prednisone. She responded within 48 hours of ritlecitinib initiation, with an immediate flare upon tapering and fast improvement upon reinitiation, confirming the efficacy of ritlecitinib. The patient has resumed normal eating and drinking habits on our treatment regimen. Her initial presentation was consistent with a desquamative gingivitis, the most common cause of which is erosive OLP. However, OLP shares overlapping features with pemphigus vulgaris, membrane pemphigus, reactive infectious mucocutaneous eruption, and herpes simplex virus, which must be ruled out before establishing an OLP diagnosis.^4^ Hepatitis may also be implicated in OLP and should be tested for during diagnostic workup.^1^^,^^3^

Diagnosing OLP is difficult and warrants thorough clinicopathologic correlation.^3^^,^^4^ The histological features observed in OLP exist on a spectrum influenced by disease stage, recent therapies, and anatomic location. It can be harder to evaluate than other OLP patterns and DIF may be necessary to establish a diagnosis. Our patient’s biopsy revealed a somewhat nonspecific mixed pattern of ulcerative lesions and reticulated involvement of the buccal cheek. However, the degree of inflammation and lymphocytic infiltration observed may have been influenced by her prednisone taper prior to her biopsy.^3^^,^^4^ The presence of Wickham striae, disease duration, negative enzyme-linked immunosorbent assay, indirect immunofluorescence, and DIF effectively ruled out other conditions and confirmed OLP.^3^^,^^4^

There is no cure for OLP, and treatments aim to provide symptomatic relief and prevent ulcer formation.^2^ First-line therapies include topical medications paired with oral hygiene practices. However, extensive disease often necessitates systemic therapy.^2^^,^^8^, ^9^, ^10^ Historically, nonspecific immunomodulators including glucocorticoids, methotrexate, azathioprine, mycophenolate mofetil, hydroxychloroquine, retinoids, and cyclosporine have been used.^2^^,^^3^ Although these may temporarily control symptoms, their side effect profiles prohibit long-term use and disease relapse after discontinuation is common.^8^, ^9^, ^10^ Thus, it is imperative to establish new OLP treatments that are effective and safe long term. While JAK inhibitors have known risks, thus far these appear to be well tolerated medications.

Biologic medications targeting specific immune pathways have recently emerged as promising OLP therapies.^9^ Because the T cell-mediated inflammation in lichen planus is mediated by JAK/signal transducer and activator of transcription signaling, JAK inhibitors may be a good therapeutic candidate.^1^^,^^2^^,^^5^^,^^7^ Select case reports and case series have demonstrated encouraging outcomes using tofacitinib, baricitinib, and upadacitinib for lichen planus disorders including OLP.^2^^,^^5^^,^^7^

The present case contributes to the growing evidence for JAK inhibition in managing OLP, specifically demonstrating potential efficacy of ritlecitinib. Unlike other JAK inhibitors primarily targeting JAK1 and/or JAK2, ritlecitinib is primarily a JAK3 and TEC kinase inhibitor.^5^^,^^7^ Given that JAK3 is upregulated in lesional epithelial cells in OLP, ritlecitinib could inhibit this process and decrease the inflammatory cascade driving OLP, as was observed in our patient.^6^

Adequate therapeutic control of OLP is paramount due to the risk of malignant transformation that occurs in 1% to 2% of adult cases.^1^^,^^2^^,^^8^^,^^9^ JAK inhibitors represent a potentially promising medication class for OLP.^7^ Although there is insufficient evidence regarding, which isoform of JAK inhibitor is most effective, all have shown some degree of clinical efficacy in alleviating symptoms and ulcer formation in OLP.^2^^,^^5^^,^^7^^,^^8^ Further research is needed to establish the true effectiveness and comparative benefits of JAK inhibitors, as well as additional classes of biologic medications for OLP. This case adds preliminary evidence for JAK3 inhibition as a treatment option for OLP and suggests that ritlecitinib could be considered for patients with refractory disease unresponsive to other systemic agents.

## Conflicts of interest

None disclosed.
